# Classification of Multiple Psychological Dimensions in Computer Game Players Using Physiology, Performance, and Personality Characteristics

**DOI:** 10.3389/fnins.2019.01278

**Published:** 2019-11-26

**Authors:** Ali Darzi, Trent Wondra, Sean McCrea, Domen Novak

**Affiliations:** ^1^Department of Electrical and Computer Engineering, University of Wyoming, Laramie, WY, United States; ^2^Department of Psychology, University of Wyoming, Laramie, WY, United States

**Keywords:** affective computing, dynamic difficulty adaptation, physiological measurements, task performance, personality characteristics, psychophysiology

## Abstract

Human psychological (cognitive and affective) dimensions can be assessed using several methods, such as physiological or performance measurements. To date, however, few studies have compared different data modalities with regard to their ability to enable accurate classification of different psychological dimensions. This study thus compares classification accuracies for four psychological dimensions and two subjective preferences about computer game difficulty using three data modalities: physiology, performance, and personality characteristics. Thirty participants played a computer game at nine difficulty configurations that were implemented via two difficulty parameters. In each configuration, seven physiological measurements and two performance variables were recorded. A short questionnaire was filled out to assess the perceived difficulty, enjoyment, valence, arousal, and the way the participant would like to modify the two difficulty parameters. Furthermore, participants’ personality characteristics were assessed using four questionnaires. All combinations of the three data modalities (physiology, performance, and personality) were used to classify six dimensions of the short questionnaire into either two, three or many classes using four classifier types: linear discriminant analysis, support vector machine (SVM), ensemble decision tree, and multiple linear regression. The classification accuracy varied widely between the different psychological dimensions; the highest accuracies for two-class and three-class classification were 97.6 and 84.1%, respectively. Normalized physiological measurements were the most informative data modality, though current game difficulty, personality and performance also contributed to classification accuracy; the best selected features are presented and discussed in the text. The SVM and multiple linear regression were the most accurate classifiers, with regression being more effective for normalized physiological data. In the future, we will further examine the effect of different classification approaches on user experience by detecting the user’s psychological state and adapting game difficulty in real-time. This will allow us to obtain a complete picture of the performance of affect-aware systems in both an offline (classification accuracy) and real-time (effect on user experience) fashion.

## Introduction

Affective games are an emerging type of videogame in which the player’s psychological (cognitive and affective) state is automatically detected and used as a basis for intelligent game adaptation (Liu et al., 2009; Chanel et al., 2011). While “classic” games perform such adaptation purely based on game performance (e.g., score), this may not obtain adequate insight into the player’s subjective state. Therefore, affective games have the potential to achieve more effective adaptation than “classic” games (Ng et al., 2012) and consequently result in higher user engagement, immersion and enjoyment (Nagle et al., 2015; McCrea et al., 2016; Denisova and Cairns, 2018). Such improvements would be useful not only for entertainment, but also for serious game applications such as education (Ip et al., 2016), motor rehabilitation (Koenig et al., 2011; Rodriguez-Guerrero et al., 2017), and autism intervention (Zhang et al., 2017b).

In an affective game, the player’s psychological state can be defined using multiple dimensions: the level of enjoyment, anxiety, or valence and arousal (Rani et al., 2004; Chanel et al., 2011; Rivas et al., 2018). Once defined, this psychological state can then be identified based on different measurements (e.g., physiology, behavioral analysis) using different machine learning (ML) methods (Novak et al., 2012). However, while some studies have compared the performance of different data modalities (e.g., physiology vs. task performance), almost all studies have only examined a single psychological dimension (e.g., only anxiety or only workload) and only one or a few similar ML approaches. There is thus only limited knowledge about how to choose the psychological dimension, data modalities, and ML approach in order to optimize psychological state estimation and game adaptation.

The goal of our study is to compare the effectiveness of different ML methods in recognizing different psychological dimensions of affective game players based on different data modalities. We thus first review data modalities (see section “Data Modalities”) and ML methods (see section “Machine Learning Methods”) used in affective games, then present the contribution of our study in more detail (see section “Contribution of This Study”).

### Data Modalities

Three data modalities are commonly used in affective games: task performance, physiological measurements, and self-assessment questionnaires. The first two are usually used as inputs to ML models that assess the level of a specific psychological dimension while questionnaires are used to obtain reference self-report levels of this psychological dimension. For example, ML models may discriminate between low and high anxiety (Liu et al., 2015), low, medium and high workload (Zhang et al., 2017a), or low and high enjoyment, with reference anxiety/workload/enjoyment values provided by questionnaires. While most applied studies focus on a single psychological dimension, it is generally acknowledged that a person’s psychological dimensions can be described using multiple dimensions simultaneously (e.g., a person can be experiencing low workload and high enjoyment). In this study, we thus define a psychological dimension as an ordinal psychological variable, and examine the ability of different data modalities to estimate multiple psychological dimensions. Below, the three data modalities are described in more detail.

#### Task Performance

Performance is a task-specific concept that is frequently used as a rough indicator of a person’s psychological state in affective computing (e.g., Williams, 2018). It varies significantly from one user to another (Salen and Zimmerman, 2004) and cannot precisely quantify the complex affects experienced during a game. Nevertheless, many studies have tried to adapt a game based on the player’s performance without assessing their psychological dimensions directly (Tan et al., 2011; Schadenberg et al., 2017; Bontchev and Georgieva, 2018). Such adaptation can have positive effects: for example, performance-based game adaptation improves children’s engagement (Bontchev and Georgieva, 2018).

#### Physiological Measures

Physiological measures from the central or peripheral nervous system can be used to quantitatively estimate psychological dimensions in a real-time manner (during the task itself) without the user’s active participation. In affective games, the most commonly used measures from the central nervous system are the electroencephalogram (EEG) (Ma et al., 2015), which records the electrical activity of the brain, and functional near infrared spectroscopy (Girouard et al., 2010), which records the hemodynamic activity associated with neural behavior. Measurements from the peripheral nervous system are largely associated with autonomic activation and include the electrocardiogram (ECG) (Rodriguez-Guerrero et al., 2017), which monitors the electrical activity of the heart (specifically heart rate), galvanic skin response (GSR) (Nourbakhsh et al., 2017), which records the activity of the skin’s sweat glands, skin temperature (ST), respiration rate (Picard et al., 2001), and others. Physiological measures are quantitative and sensitive to different kinds of stimuli, but are often affected by noise (Larson and Taulu, 2018). Most affective computing studies either use only physiology or only task performance to assess the psychological dimensions. Although a few studies have compared these two data modalities (e.g., Liu et al., 2009; Novak et al., 2011), knowledge about relative performance is limited.

#### Self-Assessment Questionnaires

Questionnaires are widely used in affective computing to assess psychological dimensions such as workload (Roscoe and Ellis, 1990), immersion (Denisova and Cairns, 2018), and emotion (Bradley and Lang, 1994). Although a self-assessment questionnaire is a reliable and accurate indicator of psychological dimensions, it cannot be used in a real-time manner since that would require pausing the task regularly.

Other types of self-assessment questionnaires can be used to provide additional information about the person that allows more accurate classification of psychological dimensions; for example, a person’s self-assessed personality could help interpret their physiological responses. Many questionnaires can be used for this purpose, including the Learning and Performance Goal Orientation Measure (Kim and Lee, 2013), behavioral inhibition/activation scales (Carver and White, 1994), self-efficiency scale (Hsia et al., 2016) and Big Five personality measures (Gosling et al., 2003). The most prominent study on this topic was performed by Johannes and Gaillard (2014), who combined personality characteristics with physiological measurements and task performance. However, as personality characteristics do not change within a game, they cannot be used as the only input to ML methods.

### Machine Learning Methods

Machine learning methods are critical in affective games, as they allow the input data (physiology, performance etc.) to be translated into an estimate of the user’s psychological dimensions, which then serves as the basis for dynamic game adaptation. In most studies, the input performance/physiological measures are classified into two, three or more levels of a psychological dimension. Previous studies have used either supervised ML methods such as linear discriminant analysis (LDA) (Chanel et al., 2011), support vector machines (SVM) (Ma et al., 2015), logistic regression (Perez et al., 2015), and artificial neural networks (Casson, 2014) or unsupervised ML methods such as Gaussian mixture models (Lee and Jung, 2006) and *k*-means clustering (Kim et al., 2009). The last few years have also seen an emerging trend of using deep learning in affective computing (Glorot et al., 2011; Jirayucharoensak et al., 2014) due to its flexibility and good performance in non-linear classification. However, deep learning requires a large dataset, which is not available in most affective game studies.

Machine learning methods for classification (Yannakakis and Hallam, 2009) or regression (Bontchev and Georgieva, 2018) can be found in all proposed real-time affective game adaptation models. However, very few have compared classification and regression on the same data with regard to their accuracy; one example was the work of Bailenson et al. (2008).

### Contribution of This Study

While most affective game studies use a single input data modality to classify one or two psychological dimensions, this study compares the accuracy of three data modalities (physiology, performance, and personality characteristics) in classification of four different psychological dimensions (perceived difficulty, enjoyment, valence, and arousal) and two subjective preferences about game difficulty (ball speed and paddle size) in a computer-based game of Pong. Our ultimate goal is to identify robust ML models that can be used to effectively adapt the difficulty of an affective game, thus ensuring an optimal game experience for the player. In this study, however, we limit ourselves to offline comparison of multiple ML techniques with different input modalities in order to find the best way to identify each psychological dimension. The research questions (RQ) are:

• RQ1: Which psychological dimension is the most sensitive to differences between several difficulty configurations? Most affective game studies focus on a single psychological dimension such as enjoyment or perceived task difficulty (Schadenberg et al., 2017) without a strong justification. A few studies, however, have examined different psychological dimensions to find the one with the highest sensitivity to the task (e.g., Baker et al., 2010). We thus hypothesized that multiple psychological dimensions can be simultaneously classified with high accuracy.
• RQ2: Which combination of ML methods and different input data modalities yields the highest classification accuracy for the different psychological dimensions and two subjective preferences regarding game difficulty? Affective games usually utilize only one data modality (e.g., performance or physiology) and compare the accuracies of different classification algorithms. Although some studies have used personality characteristics as a basis for more accurate difficulty adaptation (Nagle et al., 2016) and compared different data modalities with regard to the accuracy of ML techniques, knowledge about effective combinations of psychological dimensions, ML methods and data modalities is limited. We hypothesized that physiology will result in higher accuracy than performance or personality characteristics, but the combination of multiple data modalities will result in the most accurate classification.

## Materials and Methods

This section is divided into six subsections that describe the hardware and study setup, study protocol, physiological signals, performance variables, personality characteristics, and classification and its validation.

### Study Setup

**FIGURE 1:**
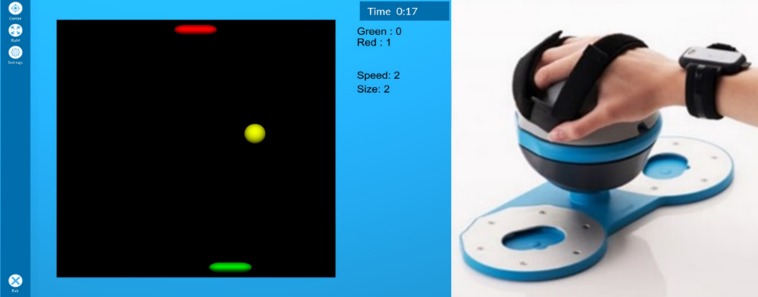
The Pong game **(left)** and the Bimeo device **(right)**. The Bimeo sits on a table and can be tilted left and right to play the game.

The computer game used in this study was reused from our previous arm rehabilitation study (Gorsic et al., 2017). It was a Pong game consisting of two paddles and a ball on a board (Figure 1, left). The bottom paddle was controlled by the participant while the top paddle was controlled by the computer. If the ball passed one player’s paddle and reached the top or bottom of the screen, the other player scored a point and the ball was instantly moved to the middle of the board, where it remained stationary for a second before moving in a random direction. The player moved their paddle left and right by tilting the Bimeo (Kinestica, Slovenia) arm tracking device (Figure 1, right) left and right with their dominant hand. While originally intended as a rehabilitation device, the Bimeo was used in order to obtain precise measurements of participants’ limb motions, which can serve as an additional input to the classifiers. The game was played on a 21-inch screen with the participant seated approximately 60 cm from the screen. Different difficulty configurations of the Pong game were used to induce different levels of psychological dimensions. The game difficulty can be adjusted using two parameters: the ball speed and the paddle size (with the paddle size being the same for both paddles at all times). This results in nine difficulty configurations, defined by all possible combinations of three ball speeds (slow, medium, and fast) and three paddle sizes (small, medium, and large).

### Study Protocol

**FIGURE 2:**
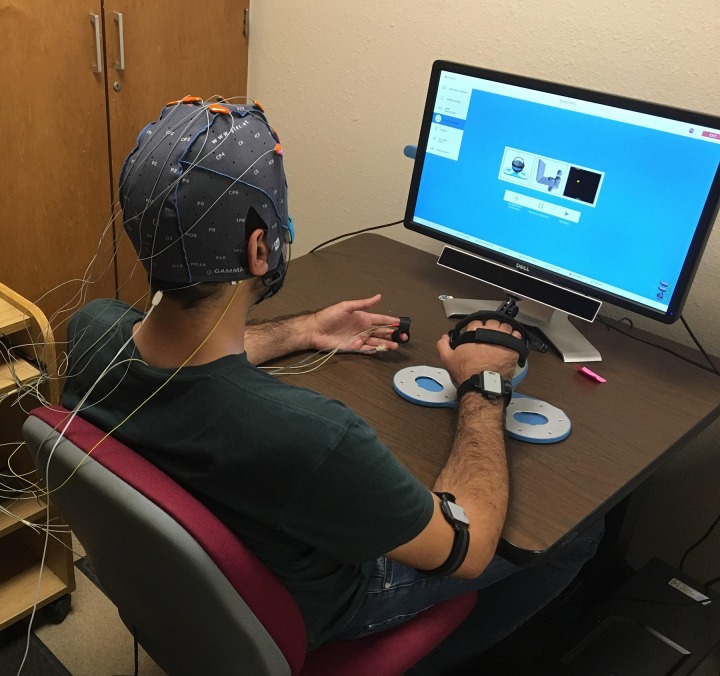
A participant relaxing during the baseline period while wearing the physiological sensors and holding the Bimeo. At the end of the baseline period, the Pong game appeared on the screen and the nine game conditions were played.

The study was approved by the University of Wyoming Institutional Review Board (protocol #2016062201232). Thirty healthy university students (24.2 ± 4.4 years old, 11 females) were recruited, and each participated in a single 1-h session. At the start of the session, participants filled out an informed consent form and four personality questionnaires. The experimenter attached all physiological electrodes; the physiological signals were then recorded for a 2-min baseline period, during which participants were instructed to relax, remain motionless and look at a basic program menu on the computer screen without closing their eyes. A photo of a participant during this baseline period is shown in Figure 2. Then, nine game conditions (corresponding to nine difficulty configurations of the Pong game) were played in random order to discourage the use of structured exploration strategies (Baranes et al., 2014). To ensure random order, 30 orders were selected using a random number generator among all possible orders of the nine difficulty configurations and presented to the participants in the order they were selected. After each 2-min condition, a short questionnaire was filled out to assess four psychological dimensions (perceived difficulty, enjoyment, valence, and arousal) and two subjective preferences about game difficulty (desired change to ball speed and paddle size). The first two psychological dimensions were assessed using simple questions (e.g., how difficult was the condition?) on 7-point scales where 1 and 7 represented very low and very high, respectively. Valence and arousal were rated using the Self-Assessment Manikin (Bradley and Lang, 1994) on a 9-point scale where 1 and 9 represented very low and very high, respectively. The range of response for desired ball speed and paddle size change was −2 to 2 where −2 means “decrease by two levels.” These responses were independent of the current ball speed and paddle size; participants could, for example, request to decrease ball speed by two levels even if it was already at the minimum value (though no participant did so). The desired changes to the ball speed and paddle size were not actually used to adapt difficulty, as the order of nine game conditions was chosen randomly for each participant before the session. The obtained results from the short questionnaire were used as the reference (response variables) for the proposed classification models. Participants’ physiological signals and performance were recorded during each condition. Signal processing and ML techniques were then applied offline using MATLAB 2016b (MathWorks, United States).

### Physiological Signals

#### Signal Acquisition and Filtering

Two g.USBamp signal amplifiers and associated sensors (g.tec Medical Engineering GmbH, Austria) were used to record six physiological signals: eight-channel EEG, two-channel electrooculogram (EOG), ECG, respiration, GSR, and ST. A similar setup was used in our recent study on physiological responses in simulated driving (Darzi et al., 2018). Eight unipolar EEG signals were recorded using g.Sahara dry electrodes (g.tec) placed on prefrontal, frontal and central areas of the brain based on the 10–20 placement system (Klem et al., 1999): AF3, AF4, F1, F2, F5, F6, C1, and C2. From these unipolar signals, four bipolar signals (AF3–AF4, F1–F2, F5–F6, C1–C2) were calculated. Two-channel EOG (reflecting up-down and left-right eye movement) was recorded from the left eye using small pre-gelled ECG electrodes (Kindall) placed according to suggestions in the literature (Ma et al., 2015). The two-channel EOG was used not only as a source of information about psychological states, but also as a reference signal with which to denoise the EEG signals (which are severely affected by eye activity). The EEG denoising was done using a recursive least squares adaptive filter with EOG as the reference (Adali and Haykin, 2010). ECG was recorded using four pre-gelled electrodes on the body (two on the chest, one over the spine, and one on the abdomen) as recommended by the manufacturer of the g.USBamp. Respiration was recorded using a thermistor-based sensor in front of the nose and mouth. ST was recorded using a small sensor attached to the distal phalanx of the little finger of the non-dominant hand using tape. GSR was recorded using two dry electrodes (g.GSRsensor2, g.tec) attached to the index and middle fingers of the non-dominant hand. Finally, a seventh physiological signal, point of gaze, was recorded using the Gazepoint GP3 remote eye tracker (Gazepoint, Canada).

The sampling frequency was 30 Hz for point of gaze and 256 Hz for all other signals. For respiration, GSR, and ST, a band-pass filter (0–30 Hz) was used to reduce high-frequency noise. For ECG, a high-pass filter (cutoff at 0.1 Hz) was used to eliminate low-frequency noise, and a 60-Hz notch filter was used to remove electrical interference. For EEG, a band-pass filter (2–60 Hz) was applied.

#### Feature Extraction

For each 2-min game condition, a total of 49 features were extracted from the seven physiological signals as follows:

*EEG*: Two methods were used: lateral power spectrum density (PSD) (Fitzgibbon et al., 2016) and dispersion entropy (Azami et al., 2017). Lateral PSD resulted in 20 features (four bipolar signals times five frequency bands) while dispersion entropy resulted in eight features (one feature per unipolar signal).

*EOG*: Mean, median and standard deviation of EOG first derivative were calculated.

*ECG*: Two time-domain features were calculated: mean heart rate and the standard deviation of inter-beat intervals. Furthermore, three frequency-domain features of heart rate variability were calculated: the power of low frequencies (LF), power of high frequencies (HF) and the power ratio of LF/HF. The LF range was 0.04–0.15 Hz while the HF range was 0.15–0.4 Hz (Shaffer and Ginsberg, 2017).

*Respiration*: The mean respiration rate (number of complete breathing cycles per minute), the standard deviation of respiration rate, and the root-mean-square of successive differences of respiration periods were calculated.

*ST*: Mean ST and the difference in ST between the first and last second of the condition were calculated.

*GSR*: The GSR can be divided into two components: the tonic (low-frequency) and phasic (high-frequency) component. For the tonic component, the mean GSR and the difference in GSR between the first and last second of the scenario were calculated. The phasic component consists of discrete skin conductance responses, and we calculated the number of responses, the mean response amplitude, and the standard deviation of response amplitude (Boucsein, 2012).

*Eye tracker*: The size of each pupil (left and right separately) and mean gaze velocity based on the point of gaze estimated by the GP3 eye tracker’s built-in software were calculated (Duchowski, 2017).

As physiological features vary widely (e.g., amplitude and frequency range) from one participant to another, they are commonly normalized to reduce intersubject differences (Novak et al., 2012). In this study, each participant’s normalized physiological features were calculated by dividing the non-normalized values by the value obtained in the baseline period. Both normalized and non-normalized versions of the physiological features were used and compared in order to find the features that would yield the highest classification accuracies.

### Performance

Two features were used to assess a participant’s performance in different game conditions: in-game score, and the amount of arm movement. The in-game score is defined as the difference of the participant’s score and the computer opponent’s score for each game condition. The amount of movement is defined as the root-mean-square value of hand velocity recorded by the Bimeo’s motion sensors, a common measure of motion intensity in arm exercise studies (Tsurumi et al., 2002). Both features were used for classification without any normalization.

### Personality Characteristics

Participants filled out four personality questionnaires: the Learning and Performance Goal Orientation Measure (Kim and Lee, 2013), Behavioral Inhibition/Activation Scales (Carver and White, 1994), the Self-efficacy Scale (Hsia et al., 2016), and the Ten Item Personality Inventory (Gosling et al., 2003). The Learning and Performance Goal Orientation Measure is a 16-item questionnaire that results in two characteristics: learning goal score and performance goal score. The Behavioral Inhibition/Activation scales questionnaire has 20 items and assesses four characteristics: behavioral inhibition, reward responsiveness, activation system drive, and fun seeking. The self-efficacy scale is a short four-item questionnaire that assesses a single characteristic: self-efficacy. The Ten Item Personality Inventory uses 10 items to assess the Big Five characteristics: extraversion, agreeableness, conscientiousness, emotional stability, and openness to experiences. All personality characteristics were used for classification without any normalization.

### Classification and Validation

All combinations of the three data modalities (physiology, performance, and personality characteristics) were used as inputs of ML methods to classify perceived difficulty, enjoyment, valence, arousal, desired change to ball speed, and desired change to paddle size (obtained from the short questionnaire) into one of multiple possible classes. The current ball speed and paddle size were added to all input data combinations since they indicate the current game state and would be available to any practical model. The reference output models for all classifiers were obtained manually from the short questionnaire. Specifically, the range of possible answers for each question on the short questionnaire was divided into either two classes, three classes, or many classes as follows.

#### Classification Into Two Classes

The input data were classified into “low” or “high” for perceived difficulty, enjoyment, valence and arousal; they were classified into “increase” or “decrease” for desired changes to paddle size and ball speed using the ranges defined in Table 1. These ranges were defined manually after data collection based on the histograms of all participants’ short questionnaire answers and ensured that the numbers of samples in each class were as equal as possible. Additionally, Table 1 presents the number of samples in each class.

**TABLE 1:** The definition of classes for two-class classification of four psychological dimensions and two subjective preferences regarding game speed and paddle size.

**Dimension**		**Difficulty**	**Enjoyment**	**Valence**	**Arousal**
Class low	Range	1–2	1–4	1–2	1–3
	# samples	86	121	93	72
Class high	Range	4–7	6–7	4–9	7–9
	# samples	123	93	120	95

**Preference**		**Speed change**		**Paddle size change**	

Class Decrease	Range	−1, 0		−2, −1	
	# samples	120		79	
Class Increase	Range	1, 2		1, 2	
	# samples	150		60	

*The classes are defined based on histograms of possible answers to that question on the short questionnaire. # samples = number of samples in class.*

#### Classification Into Three Classes

The input data were classified similarly to the above scenario, but the possible classes were now low/medium/high (for perceived difficulty, enjoyment, valence, and arousal) or increase/decrease/no change (for desired changes to paddle size and ball speed) using the ranges defined in Table 2. Again, these ranges were manually defined based on histograms of participants’ answers. Additionally, Table 2 presents the number of samples in each class.

**TABLE 2:** The definition of classes for three-class classification of four psychological dimensions and two subjective preferences regarding game speed and paddle size.

**Dimension**		**Difficulty**	**Enjoyment**	**Valence**	**Arousal**
Class low	Range	1–2	1–3	1–2	1–3
	# samples	86	76	93	72
Class medium	Range	3–4	4–5	3–4	4–6
	# samples	101	101	95	103
Class high	Range	5–7	6–7	5–9	7–9
	# samples	83	93	82	95

**Preference**		**Speed change**		**Paddle size change**	

Class Decrease	Range	−2, −1		−2, −1	
	# samples	13		79	
Class No change	Range	0		0	
	# samples	107		131	
Class Increase	Range	1, 2		1, 2	
	# samples	150		60	

*The classes are defined based on histograms of possible answers to that question on the short questionnaire. # samples = number of samples in class.*

#### Classification Into Many Classes

Unlike the previous two scenarios, the answers to the short questionnaire were not mapped to two or three classes; instead, the number of classes for each outcome variable was the same as the number of possible answers to that question on the short questionnaire. Thus, the input data were classified into seven possible classes for perceived difficulty and enjoyment (which had a range of 1–7 on the short questionnaire), nine classes for valence and arousal, and five for the desired changes to ball speed and paddle size. This scenario is henceforth referred to as “many classes” to be concise. Two-class and three-class classification are common in affective games. Classification into “many classes,” on the other hand, is not common, but was added to evaluate the possibility of high-resolution classification as well as allow direct impartial comparison of regression to other classifiers. Since regression considers the numerical relationship between the classes while other classifiers do not, it was expected to be more accurate for classification into many classes.

As the basis for all classification scenarios, we first used forward stepwise feature selection (Keough and Quinn, 1995) on the full dataset to find the most informative set of features. The inclusion threshold for feature selection was 0.05 for the two- and three-class scenarios; it was 0.1 for the “many classes” scenario. Then, all combinations of the three data modalities (performance, physiology, and personality characteristics) were classified using four different classifiers: SVM with a linear kernel, LDA, ensemble decision tree, and multiple linear regression. The classification was done for each outcome variable of the short questionnaire (perceived difficulty, enjoyment, valence, arousal, desired change to ball speed, and paddle size) and each classification scenario separately. To use multiple linear regression as a classifier, its continuous output value was rounded to the closest class. The classifiers were validated using 10-fold crossvalidation (27 participants’ data used to train, three participants’ data to validate the classifier; procedure repeated 10 times with each participant in the validation dataset once).

## Results

### Classification

Table 3 presents the mean two-class classification accuracies for all combinations of input data modalities. The highest accuracy (97.6%) was obtained for classification of desired changes to paddle size using only physiological measurements. Physiological measurements alone yielded the highest accuracy for four of six outcome variables; for the other two, the highest accuracy was obtained with the combination of all data modalities. The lowest classification accuracy (89.3%) was obtained for desired changes to ball speed. Table 3 presents the results of only the most accurate of the four classifiers in each classification scenario; accuracies for all four classifiers are presented in Supplementary Table S4. Standard deviations of these classification accuracies are available in Supplementary Table S1.

**TABLE 3:** Mean two-class classification accuracies for all combinations of input data modalities.

	**Outcome variables**					
**Input data modality**	**Difficulty**	**Enjoyment**	**Valence**	**Arousal**	**Speed change**	**Paddle size change**
Physiology	^N^94.3% (R)	86.3% (S)	**^N^95.3% (R)**	**^N^95.8% (R)**	**89.3% (R)**	**^N^97.6% (S)**
Personality	84.7% (E)	83.4% (E)	84.2% (E)	87.4% (E)	81.4% (E)	92.5% (S)
Performance	84.3% (R)	68.0% (S)	62.3% (E)	77.8% (E)	75.5% (L)	92.1% (L)
Physio and Pers	^N^95.2% (R)	^N^92.1% (R)	93.0% (R)	^N^95.4% (R)	^N^88.2% (S)	96.5% (S)
Physio and Perf	^N^94.7% (R)	^N^94.4% (R)	^N^94.9% (R)	^N^95.2% (S)	87.4% (R)	97.1% (S)
Pers and Perf	85.2% (E)	82.4% (E)	83.3% (E)	87.3% (E)	82.7% (E)	92.1% (S)
All	**^N^96.2% (R)**	**^N^96.3% (R)**	93.9% (R)	^N^95.5%(R)	^N^87.6% (S)	96.8% (S)

*The highest accuracy in each column is bolded. Physio, Physiology; Pers, Personality characteristics; Perf, Performance; S, Support vector machine; L, linear discriminant analysis; E, ensemble decision tree; R, multiple linear regression; ^*N*^, Normalized physiological features.*

Table 4 presents the mean three-class classification accuracies for all combinations of the three data modalities. The highest classification accuracy was obtained for desired changes to ball speed and paddle size (84.1%) while the lowest was obtained for arousal level (73.3%). The combination of physiological measurements and personality characteristics yielded the highest classification accuracy for four of six outcome variables; for the other two, the combination of all data modalities resulted in the highest accuracy. Standard deviations of these classification accuracies are available in Supplementary Table S2.

**TABLE 4:** Mean three-class classification accuracies for all combinations of input data modalities.

	**Outcome variables**					
**Input data modality**	**Difficulty**	**Enjoyment**	**Valence**	**Arousal**	**Speed change**	**Paddle size change**
Physiology	^N^76.3% (R)	^N^70.0% (R)	65.6% (S)	^N^69.6% (R)	^N^83.7% (R)	^N^83.7% (S)
Personality	63.3% (E)	60.4% (E)	62.6% (E)	68.5% (E)	78.2% (S)	73.0% (E)
Performance	59.6% (S)	47.1% (S)	41.5% (S)	56.0% (S)	71.1% (E)	58.5% (S)
Physio and Pers	**^N^81.5% (R)**	**^N^75.2% (R)**	72.2% (S)	^N^72.2% (R)	**^N^84.1%(R)**	**^N^84.1% (S)**
Physio and Perf	^N^77.1% (R)	^N^74.4% (R)	^N^66.1% (R)	67.8% (L)	^N^83.7% (R)	^N^83.0% (S)
Pers and Perf	62.6% (E)	63.3% (E)	61.8% (E)	63.0% (L)	79.6% (E)	71.1% (E)
All	^N^76.7% (R)	^N^70.0% (R)	**74.4% (R)**	**^N^73.3% (R)**	**^N^84.1% (S)**	^N^81.5% (S)

*The highest accuracy in each column is bolded. Physio, Physiology; Pers, Personality characteristics; Perf, Performance; S, Support vector machine; L, linear discriminant analysis; E, ensemble decision tree; R, multiple linear regression; ^*N*^, Normalized physiological features.*

Table 5 presents the mean “many classes” classification accuracies for all combinations of the three data modalities. In this scenario, there were seven classes for perceived difficulty and enjoyment, nine for valence and arousal, and five for changes to ball speed and paddle size. The highest classification accuracy was obtained for speed and paddle size change (approximately 65%) while the lowest was obtained for arousal (approximately 30%). The combination of all data modalities yielded the highest accuracy for five of six classification cases; for the other one, the physiological measurements or the combination of physiology and performance yielded the highest accuracy. Standard deviations of these classification accuracies are available in Supplementary Table S3.

**TABLE 5:** Mean “many-class” classification accuracies for all combinations of input data modalities.

	**Outcome variables**					
	**7 classes**		**9 classes**		**5 classes**	
**Input data modality**	**Difficulty**	**Enjoyment**	**Valence**	**Arousal**	**Speed change**	**Paddle size change**
Physiology	37.4% (R)	31.5% (S)	**^N^38.9% (S)**	28.6% (S)	^N^60.0% (R)	58.5% (S)
Personality	34.1% (S)	**38.2% (E)**	36.3% (E)	23.3% (S)	60.6% (E)	61.8% (S)
Performance	34.8% (E)	27.1% (R)	23.3% (S)	18.9% (R)	53.0% (L)	47.5% (L)
Physio and Pers	^N^35.6% (R)	37.1% (S)	^N^34.8% (S)	26.7% (S)	**65.2% (R)**	65.6% (S)
Physio and Perf	37.4% (R)	^N^37.4% (S)	**^N^38.9% (S)**	^N^25.6% (S)	^N^60.0% (R)	60.7% (S)
Pers and Perf	34.1% (S)	35.2% (S)	31.1% (E)	25.6% (E)	63.3% (E)	63.7% (E)
All	**38.8% (R)**	**^N^38.2% (S)**	^N^35.6% (S)	**^N^29.5% (S)**	**65.2% (R)**	**66.3% (E)**

*The highest accuracy in each column is bolded. Physio, Physiology; Pers, Personality characteristics; Perf, Performance; S, Support vector machine; L, linear discriminant analysis; E, ensemble decision tree; R, multiple linear regression; ^*N*^, Normalized physiological features.*

### Best Selected Features

Table 6 shows the best selected features for two-class classification of the six outcome variables from the short questionnaire: perceived difficulty, enjoyment, valence, arousal, desired change to ball speed, and paddle size. The “best” features are considered to be the first four features selected by forward stepwise feature selection among all features from all three data modalities. Each feature’s mean value and standard deviation are shown separately for each class (e.g., low or high); furthermore, the significance of each feature’s differences between the two classes is indicated with *P*-values.

**TABLE 6:** The best four features chosen by stepwise feature selection for each outcome variable.

**Outcome variable**	**Rank**	**Selected feature**	***P*-value**	**Class low (Mean ± SD)**	**Class high (Mean ± SD)**
Difficulty	1	Current speed (1.5–3.5)	<0.001	2.4 ± 0.5	3.4 ± 0.7
	2	Current paddle size (1–3)	<0.001	2.2 ± 0.8	1.7 ± 0.8
	3	Lateral PSD of AF3/AF4 in Gamma band	<0.001	0.11 ± 0.93	−0.15 ± 0.95
	4	Behavioral inhibition (7–28)	0.01	19.9 ± 3.4	18.9 ± 3.8
Enjoyment	1	Current speed (1.5–3.5)	<0.001	2.8 ± 0.8	3.2 ± 0.8
	2	Learning goal (8–56)	<0.001	48.1 ± 6.0	50.8 ± 4.3
	3	In-game score	<0.001	3.6 ± 7.1	6.2 ± 7.4
	4	Arm movement level	<0.001	−0.12 ± 0.76	0.18 ± 0.95
Valence	1	Normalized left pupil size	<0.001	−0.03 ± 0.73	0.51 ± 1.07
	2	Learning goal (8–56)	<0.001	48.0 ± 6.3	51.1 ± 3.9
	3	Agreeableness (2–14)	<0.001	5.7 ± 1.6	6.6 ± 1.8
	4	Lateral PSD of C1/C2 in Gamma band	<0.001	0.25 ± 0.93	−0.12 ± 0.82
Arousal	1	Current speed (1.5–3.5)	<0.001	2.5 ± 0.7	3.3 ± 0.8
	2	Normalized eye movement velocity	<0.001	−0.13 ± 0.67	0.77 ± 1.25
	3	Current paddle size (1–3)	<0.001	2.3 ± 0.1	1.8 ± 0.1
	4	Normalized mean respiration rate	0.001	0.04 ± 0.95	0.28 ± 0.81
	**Rank**	**Selected feature**	***P*-value**	**Decrease (Mean ± STD)**	**Increase (Mean ± STD)**
Speed change	1	Current speed (1.5–3.5)	<0.001	3.5 ± 0.7	2.6 ± 0.7
	2	Self-efficacy (4–20)	<0.001	15.1 ± 2.0	15.9 ± 2.4
	3	Openness to experience (2–14)	0.002	10.8 ± 1.9	10.6 ± 2.0
	4	Dispersion entropy of AF3 (−1 to 1)	0.003	−0.16 ± 0.84	0.11 ± 1.06
Paddle size change	1	Current paddle size (1–3)	<0.001	2.7 ± 0.1	1.2 ± 0.1
	2	Extraversion (2–14)	<0.001	7.4 ± 3.1	8.7 ± 2.7
	3	Agreeableness (2–14)	<0.001	6.6 ± 1.9	5.8 ± 1.5
	4	Dispersion entropy of C2 (−1 to 1)	0.001	−0.19 ± 1.04	0.13 ± 0.90

*The features are selected for the case of two-class classification among all features from all three data modalities. For questionnaires and current difficulty settings, the minimum and maximum possible value are given. For all features, the mean **±** standard deviation are given for the feature’s value in each class. The significance level of the difference between the two classes is shown with *P*-values. PSD, power spectrum density; SD, standard deviation.*

## Discussion

### Classification of Different Psychological Dimensions

Tables 3–5 indicate that two-class classification is most effective using physiological measurements, three-class classification is most effective using a combination of physiology and personality characteristics, and many-class classification is most effective using a combination of all three data modalities. Thus, physiological measurements are more informative than performance and personality data and should be collected despite the relatively high cost and difficulty of measurement compared to performance measurements.

Though physiological data generally exhibited the highest classification accuracy, the accuracies of performance and personality data were not worse than the physiological ones by more than 15%, which was somewhat unexpected – we expected that personality data would be nearly useless on their own. Further analysis revealed that the high accuracy was because the current speed and current paddle size were included in all classifiers. As a follow-up evaluation, all classification cases were repeated without adding the current game parameters to the input data, resulting in a large decrease in all classification accuracies. For two-class classification, the largest decrease was observed for perceived difficulty (96–66%) while the smallest was observed for valence (95–84%). For three-class classification, the largest decrease was again observed for perceived difficulty (81–46%) while the smallest was observed for valence (74–63%). For many-class classification, the largest decrease was observed for desired paddle size change (66–50%) while the smallest was observed for valence (39–37%). Therefore, the current state of the game already allows some estimation of how the user is likely to want to adjust difficulty, and this should be taken into account in general affective computing research.

Based on Tables 3–5, perceived difficulty is the most promising psychological dimension for use in a real-time application, as it is classified more accurately than the other three dimensions. Furthermore, it is the only one that can be directly used as a basis for adaptation – if-then rules can easily be designed to adapt the difficulty parameters based on perceived difficulty. Enjoyment, for example, provides a less intuitive basis for adaptation, as low enjoyment could be caused by the game being either too easy or too hard. Other than the psychological dimensions, the two subjective preferences regarding the difficulty parameters could also be accurately predicted in a real-time application using the developed classifiers, and could provide more fine-grained information about how exactly difficulty should be adapted.

### Machine Learning Methods

According to Tables 3–5, regression and SVM are the most accurate classifiers. In two-class classification, regression is the dominant classifier, however, in three- and many-class classification, other classifiers (especially SVM) are also often the most accurate. In a practical application, developers could mix and match classifiers and data modalities, choosing whatever approach is most accurate for each specific goal (e.g., use one approach for classification of desired ball speed change and a different approach for desired paddle size change).

In 65% of classification cases that involved physiological features (either alone or with personality/performance), the normalized physiological features resulted in higher accuracy than non-normalized ones. Furthermore, in 66% of classification cases with normalized physiological features, the regression-based classifier was more accurate than the other three classifiers. Therefore, since normalization is computationally not demanding, it should be used in the classification cases where it improves accuracy; when normalization is used, regression-based classification is most likely to be effective.

As the specific classes (low, medium, and high) were defined for each psychological dimension and subjective preference based on the histogram of responses, the number of samples in different classes is similar between dimensions and preferences, and should not have a major effect on relative classification accuracies. The one exception to this is in the “many classes” case, where the subjective preferences yield higher accuracies due to a lower number of possible classes (five vs. seven or nine for psychological dimensions).

### The Best Selected Features

Table 6 lists the best four selected features for two-class classification of all short questionnaire outputs (perceived difficulty, enjoyment, valence, arousal, desired speed, and paddle size change). These features were selected by the stepwise method; however, the causal relationship between the features and the output is unclear and beyond the scope of this paper. Below, we discuss the best selected features for each questionnaire output.

#### Perceived Difficulty

The current speed and paddle size values are the most effective features, showing that the game’s difficulty parameters are effective. Lateral PSD of prefrontal electrodes (AF3/AF4) decreases during difficult game conditions, indicating a lower activation of the right prefrontal lobe. Behavioral inhibition predicts an individual’s response to anxiety-relevant cues, and higher values indicate more intense inhibition. The obtained results show that behavioral inhibition is lower among the participants who reported higher perceived difficulty.

#### Enjoyment

Current ball speed was the best predictor of enjoyment, indicating that participants enjoyed themselves more when the game was harder. However, this result is likely specific to our participants (mostly young university students); furthermore, it is likely specific to the range of tested ball speeds, as higher difficulties would likely cause enjoyment to drop again. Learning goal, a personality characteristic, is also a predictor of enjoyment. We consider this reasonable, as this characteristic indicates an individual’s persistence in learning; thus, participants who are more persistent likely learn how to play the game better and enjoy themselves more.

#### Valence

Participants’ pupil size is significantly larger when they experience more positive emotions. Previous studies have indicated that pupil size can increase with both positive and negative stimuli, supporting this finding (Partala and Surakka, 2003). Furthermore, two personality characteristics are correlated with valence: agreeableness and learning goal. As with enjoyment, participants with a higher learning goal score may have been more persistent in learning how to play; participants with high agreeableness, on the other hand, may have been simply more likely to exhibit higher enjoyment in general. Finally, the lateral PSD of the central electrodes (C1/C2) decreases as valence increases.

#### Arousal

The current ball speed and paddle size act as predictors of arousal, which is unsurprising – higher difficulties require more arousal. Furthermore, eye movement velocity increases with arousal, likely since participants need to track the moving ball and paddles across the screen more quickly. The general trend of eye velocity being correlated with arousal is also seen in other affective computing studies (e.g., Di Stasi et al., 2013). Furthermore, respiration rate increases with arousal, which could be either due to psychological effects or due to the higher physical demand associated with faster arm movements.

#### Desired Speed Change

The current speed is a predictor of how participants would like to change the speed, which can be considered a trivial result. More interestingly, self-efficacy, which assesses the personal judgment of “how well one can execute courses of action required to deal with prospective situations,” is higher in participants who prefer to increase the speed and make the game harder. Furthermore, the dispersion entropy for the AF3 electrode is higher when participants prefer to increase speed.

#### Desired Paddle Size Change

Again, the current paddle size is a trivial predictor of desired paddle size changes. More interestingly, two personality changes are predictors of desired paddle size changes: participants with high extraversion prefer to increase paddle size while participants with high agreeableness prefer to decrease it. However, the reason for this relationship is unclear. Finally, the dispersion entropy for the C2 electrode is lower when participants prefer to decrease paddle size. Similar correlations between EEG dispersion entropy and mental workload have been found in other studies (e.g., Zarjam et al., 2015).

### Next Steps

As the classifiers are highly accurate, our next step will be to use them in a real-time manner: the participant’s psychological dimension will be classified, the game will adapt its difficulty based on this information, and the effect on the overall user experience will be evaluated. We consider such real-time evaluation to be critical, as differences in offline classification accuracy may not actually translate to differences in actual user experience (Novak et al., 2014; McCrea et al., 2016).

Since the classifiers are not computationally demanding, real-time versions have already been made and preliminarily tested. In the future, we will evaluate ways to increase their accuracy by also providing them with information about previous difficulty configurations experienced by the specific participant and the evoked psychological dimensions. This would allow the affective computing system to estimate how a specific participant is likely to react to a certain difficulty configuration based on past data, providing personalized classification.

At the same time, as our ultimate goal is to enable the development of practical affective game systems, we should consider how the approach can be simplified for real-world use. None of the classifiers used in our work are computationally demanding, and both task performance and personality measures can be obtained easily. The main obstacle is the use of laboratory-grade sensors that take time to apply. In the future, we will thus also evaluate less obtrusive sensors such as heart rate sensors embedded in the game controller (Abe et al., 2015) or eye trackers embedded in head-mounted virtual reality systems (Hua, 2001).

### Study Limitations

Three limitations of the study should be acknowledged. First, we performed classification over 2-min intervals so that results of our study could be compared to those of other studies on physiology-based analysis of psychological dimensions, which tend to use intervals of 2–5 min. However, shorter or longer intervals could result in different classification accuracies, and both shorter and longer intervals have their advantages: shorter intervals would allow difficulty to be adapted more often while longer intervals would allow more complex emotions to be induced.

Second, the training data were limited to nine difficulty configurations (three speeds and three paddle sizes), and these nine configurations were not perceived as overwhelmingly difficult by most participants. This is indicated by participants’ opinions about desired difficulty changes, as they were more likely to want to increase rather than decrease difficulty. As a consequence, the full range of emotion that could be experienced in an affective game was likely not induced – for example, most participants did not experience very high perceived difficulty or frustration. We have partially compensated for this shortcoming by redefining the class ranges manually based on histograms of participants’ responses to the short questionnaire, which ensures that the dataset still contains a relatively balanced distribution of samples from “low,” “medium,” and “high” classes. Still, it must be acknowledged that this distribution reflects the range of emotion induced by our study protocol, not the range of emotion induced by all affective games. Thus, the developed classifiers may not generalize to more extreme difficulty configurations of our game or to other games that induce different levels and types of workload. For example, our game primarily induces temporal workload due to the need to quickly intercept the ball, and the developed classifiers may not generalize to games that primarily induce mental workload in the absence of temporal workload (e.g., a math practice game with no time limits). However, this is not simply a limitation of our study, as generalizability of results between tasks has long been a challenge in affective computing.

Finally, the study was conducted with a sample of healthy university students who were mostly Caucasian and had prior experience with computer games. Thus, care should be taken when generalizing the results to other populations. Older or non-Caucasian participants, for example, may have both different physiological responses to stress and different psychological reactions to computer games.

## Conclusion

In this study, four psychological dimensions and two subjective preferences of participants who played Pong at nine difficulty configurations were classified into either two, three or many classes using four classifiers: SVM, LDA, ensemble decision tree and multiple linear regression. Reference class labels were defined based on participants’ answers to a short questionnaire after each game condition. The classifiers used different combinations of three input data modalities (physiological measurements, game performance, and personality characteristics), and classification accuracies were compared between data modalities.

The highest classification accuracies were 97.6% for two-class classification, 84.1% for three-class classification, and 66.3% for “many classes” classification. The four psychological dimensions exhibited different classification accuracies, with the highest accuracy achieved for perceived difficulty. As perceived difficulty is also the easiest to use as a basis for difficulty adaptation, we thus recommend using it if the goal is to adapt game difficulty based on psychological dimensions. The two subjective preferences about game difficulty were also highly accurate and could be used instead of or in addition to the overall perceived difficulty estimate. Physiological measurements were the most informative data modality and are thus worth including despite the additional hardware and preparation time; furthermore, they should ideally be normalized, as normalization increased classification accuracy in the majority of cases. However, other data modalities should not be ignored, as the current game difficulty, personality characteristics and game performance also usefully contributed to classification accuracy. Table 6 specifically lists the most useful features for classification of the different psychological dimensions and subjective preferences, providing guidance for developers of affective games.

As the next step, we will use our classifiers in a real-time manner: to adapt game difficulty based on the participant’s psychological dimensions. We will then study the effects of different classification methods on user enjoyment and game performance, allowing us to obtain a complete picture of the performance of affective games in both an offline (classification accuracy) and real-time (effect on user experience) fashion. In the long term, comparisons of different methods will allow us to identify the most effective ways to increase participant engagement in an affective game, resulting in an improved user experience. In the case of serious games (e.g., rehabilitation games), increased engagement may also lead to improved game outcomes – e.g., higher exercise intensity in a rehabilitation game or improved learning rate in an educational game.

## Data Availability Statement

The data supporting the conclusion of this manuscript are included as a Supplementary File. The file contains the different features (physiology, performance, and personality characteristics) for all participants and game conditions as well as the results of the short questionnaire. Both raw (non-normalized) and normalized features are provided as Supplementary Tables S5, S6 on separate sheets. To protect participant anonymity, potentially identifiable information (age, gender, dominant hand, etc.) have been omitted.

## Ethics Statement

The study was carried out in accordance with the recommendations of the Belmont Report and the Collaborative Institutional Training Initiative, with written informed consent from all subjects. All subjects gave written informed consent in accordance with the Declaration of Helsinki. The protocol was approved by the University of Wyoming Institutional Review Board (protocol #2016062201232).

## Author Contributions

AD led the data collection and analysis, and wrote the majority of the manuscript. TW and SM assisted with the literature review, study design, and data analysis. DN supervised the entire study, led the study design, and contributed to the data analysis and manuscript writing. All authors read and approved the final version of the manuscript.

## Conflict of Interest

The authors declare that the research was conducted in the absence of any commercial or financial relationships that could be construed as a potential conflict of interest.
