# Supplementary material for: Classification of Multiple Psychological Dimensions in Computer Game Players Using Physiology, Performance, and Personality Characteristics
Source: Front Neurosci. 2019 Nov 26;13:1278. doi: 10.3389/fnins.2019.01278 (PMC6888016; doi:10.3389/fnins.2019.01278)
Supplement: Supplementary file 1 [file Table_1.docx]

Supplementary Material

**Classification and Regression of Multiple Psychological Dimensions in Computer Game Players Using Physiology, Performance and Personality Characteristics**

**Ali Darzi, Trent Wondra, Sean McCrea, and Domen Novak**

*** Correspondence:** Domen Novak: [dnovak1@uwyo.edu](mailto:dnovak1@uwyo.edu)

# Raw data

The raw data supporting the conclusions of this manuscript are included as a Microsoft Excel (.xls) spreadsheet. The file contains the different features (physiology, performance, and personality characteristics) for all participants and game conditions as well as the results of the short questionnaire. Both raw (non-normalized) and normalized features are provided as Tables S.5 and S.6 on separate sheets. To protect participant anonymity, potentially identifiable information (age, gender, dominant hand, etc.) have been omitted.

# Standard deviations of classification accuracies

The main paper presents the mean classification accuracies for all combinations of data modalities in Tables 1-3. However, as this does not provide any information about variability, this supplementary material provides the corresponding standard deviations of the classification accuracies. Each of the tables below directly corresponds to a table in the main paper; for example, Table S.1 corresponds to Table 1, and each cell in Table S.1 contains the standard deviation corresponding to the mean value in the same cell of Table 1.

# Table S.1. Standard deviations of two-class classification accuracies for all combinations of input data modalities. The standard deviation associated with the highest mean classification accuracy in each column is bolded. Physio: Physiology, Pers: Personality characteristics, Perf: Performance, S: Support vector machine, L: linear discriminant analysis, E: ensemble decision tree, R: multiple linear regression, ^N^: Normalized physiological features.

|  | Outcome variables | | | | | |
| --- | --- | --- | --- | --- | --- | --- |
| Input data modality | Difficulty | Enjoyment | Valence | Arousal | Speed change | Paddle size Change |
| Physiology | ^N^9.2% (R) | 9.4% (S) | **^N^4.6% (R)** | **^N^4.8% (R)** | **7.1% (R)** | **^N^4.6% (S)** |
| Personality | 12.8% (E) | 10.6% (E) | 7.8% (E) | 7.8% (E) | 7.4% (E) | 9.0% (S) |
| Performance | 14.4% (R) | 11.6% (S) | 10.3% (E) | 9.3% (E) | 12.4% (L) | 11.4% (L) |
| Physio & Pers | ^N^7.4% (R) | ^N^4.5% (R) | 7.2% (R) | ^N^4.8% (R) | ^N^6.9% (S) | 4.0% (S) |
| Physio & Perf | ^N^9.1% (R) | ^N^4.0% (R) | ^N^7.1% (R) | ^N^5.8% (S) | 6.5% (R) | 3.6% (S) |
| Pers & Perf | 12.1% (E) | 9.5% (E) | 6.6% (E) | 8.4% (E) | 7.1% (E) | 9.2% (S) |
| All | **^N^5.ƒ8% (R)** | **^N^6.1% (R)** | 7.1% (R) | ^N^4.8% (R) | ^N^4.1% (S) | 3.7% (S) |

**Table S.2.** Standard deviations of three-class classification accuracies for all combinations of input data modalities. The standard deviation associated with the highest mean classification accuracy in each column is bolded. Physio: Physiology, Pers: Personality characteristics, Perf: Performance, S: Support vector machine, L: linear discriminant analysis, E: ensemble decision tree, R: multiple linear regression, ^N^: Normalized physiological features.

|  | Outcome variables | | | | | |
| --- | --- | --- | --- | --- | --- | --- |
| Input data modality | Difficulty | Enjoyment | Valence | Arousal | Speed change | Paddle size Change |
| Physiology | ^N^10.4% (R) | ^N^8.4% (R) | 11.2% (S) | ^N^10.7% (R) | ^N^7.2% (R) | ^N^5.2% (S) |
| Personality | 11.2% (E) | 17.3% (E) | 8.3% (E) | 6.8% (E) | 10.4% (S) | 6.3% (E) |
| Performance | 15.4% (S) | 9.3% (S) | 10.4% (S) | 11.1% (S) | 13.1% (E) | 8.0% (S) |
| Physio & Pers | ^N^**7.4% (R)** | **^N^6.5% (R)** | 9.6% (S) | ^N^7.5% (R) | **^N^5.8% (R)** | **^N^7.5% (S)** |
| Physio & Perf | ^N^11.3% (R) | ^N^7.3% (R) | ^N^11.3% (R) | 9.7% (L) | ^N^7.2% (R) | ^N^5.8% (S) |
| Pers & Perf | 9.2% (E) | 15.5% (E) | 5.9% (E) | 12.3% (L) | 9.2% (E) | 11.9% (E) |
| All | ^N^11.0% (R) | ^N^6.0% (R) | **7.6% (R)** | ^N^**12.3% (R)** | **^N^9.1% (S)** | ^N^7.2% (S) |

**Table S.3.** Standard deviations of “many-class” classification accuracies for all combinations of input data modalities. The standard deviation associated with the highest mean classification accuracy in each column is bolded. Physio: Physiology, Pers: Personality characteristics, Perf: Performance, S: Support vector machine, L: linear discriminant analysis, E: ensemble decision tree, R: multiple linear regression, ^N^: Normalized physiological features.

|  | Outcome variables | | | | | |
| --- | --- | --- | --- | --- | --- | --- |
|  | **7 classes** | | **9 classes** | | **5 classes** | |
| Input data modality | Difficulty | Enjoyment | Valence | Arousal | Speed change | Paddle size Change |
| Physiology | 12.6% (R) | 10.2% (S) | **^N^6.6% (S)** | 6.8% (S) | ^N^5.2% (R) | 9.5% (S) |
| Personality | 8.1% (S) | **13.3% (E)** | 8.8% (E) | 7.5% (S) | 8.9% (E) | 8.9% (S) |
| Performance | 12.3% (E) | 7.6% (R) | 7.6% (S) | 5.9% (R) | 13.0% (L) | 10.7% (L) |
| Physio & Pers | ^N^8.7% (R) | 8.2% (S) | ^N^9.9% (S) | 8.5% (S) | **14.1% (R)** | 11.2% (S) |
| Physio & Perf | 12.6% (R) | ^N^13.2% (S) | **^N^7.0% (S)** | ^N^8.3% (S) | ^N^8.7% (R) | 7.2% (S) |
| Pers & Perf | 8.1% (S) | 7.9% (S) | 4.7% (E) | 4.4% (E) | 9.3% (E) | 9.8% (E) |
| All | **9.9% (R)** | **^N^10.1% (S)** | ^N^8.9% (S) | **^N^8.4% (S)** | **14.1% (R)** | **7.5% (E)** |

# Two-class classification accuracies for all four classifiers separately

Table 1 in the main paper presents the best obtained accuracies for two-class classification using four classifiers: support vector machine, linear discriminant analysis, ensemble decision tree, multiple linear regression. To demonstrate the differences in performance between these four classifiers, Table S.4 is an expanded version of Table 1 that shows accuracies for all four classifiers.

# Table S.4. The obtained classification accuracies for all four classification methods. The highest classification accuracies among the four classifiers in each column are bolded. SVM: Support vector machine, LDA: linear discriminant analysis, EDT: ensemble decision tree, ^N^: Normalized physiological features.

| Outcome Accuracies | | | | | | | |
| --- | --- | --- | --- | --- | --- | --- | --- |
| Input data modality | Classification Method | Difficulty | Enjoyment | Valence | Arousal | Speed change | Paddle size Change |
| Physiology | SVM | 91.6% | **86.3%** | 88.6% | ^N^93.5% | 87.1% | **^N^97.6%** |
|  | LDA | 78.8% | 76.5% | 76.3% | 74.0% | 75.6% | 73.1% |
|  | EDT | ^N^83.2% | 76.8% | 79.3% | 84.2% | 79.1% | 93.7% |
|  | Regression | **^N^94.3%** | 86.5% | **^N^95.3%** | **^N^95.8%** | **89.3%** | ^N^87.8% |
| Personality | SVM | 84.1% | 72.2% | 75.2% | 80.8% | 79.2% | **92.5%** |
|  | LDA | 77.1% | 66.5% | 66.6% | 72.7% | 76.8% | 91.4% |
|  | EDT | **84.7%** | **83.4%** | **84.2%** | **87.4%** | **81.4%** | 92.1% |
|  | Regression | 83.7% | 70.0% | 78.0% | 81.4% | 80.0% | 70.5% |
| Performance | SVM | 82.1% | **68.0%** | 61.0% | 76.9% | 72.8% | 91.4% |
|  | LDA | 76.4% | 62.8% | 57.0% | 82.1% | **75.5%** | **92.1%** |
|  | EDT | 78.5% | 65.1% | **62.3%** | **77.8%** | 74.3% | 90.6% |
|  | Regression | **84.3%** | 67.3% | 60.1% | 77.3% | 74.1% | 74.1% |
| Physiology & Personality | SVM | ^N^91.0% | ^N^85.3% | 88.7% | 91.0% | **^N^88.2%** | **96.5%** |
|  | LDA | 76.5% | 63.5% | 67.0% | 66.9% | 75.6% | 67.1% |
|  | EDT | 83.2% | 79.6% | 83.3% | 87.4% | 79.4% | ^N^92.2% |
|  | Regression | **^N^95.2%** | **^N^92.1%** | **93.0%** | **^N^95.4%** | ^N^87.0% | ^N^94.2% |
| Physiology & Performance | SVM | ^N^93.4% | 88.6% | 87.6% | 87.7% | ^N^86.7% | **97.1%** |
|  | LDA | 77.8% | 56.5% | 76.3% | 66.9% | 78.0% | 66.8% |
|  | EDT | 81.1% | 82.2% | 76.6% | 83.5% | ^N^78.2% | ^N^97.3% |
|  | Regression | **^N^94.4%** | **^N^94.4%** | **94.9%** | **^N^95.2%** | **87.4%** | 86.3% |
| Personality & Performance | SVM | 83.8% | 78.8% | 76.7% | 80.8% | 79.6% | **92.1%** |
|  | LDA | 77.3% | 67.5% | 62.6% | 70.1% | 78.7% | 91.4% |
|  | EDT | **85.2%** | **82.4%** | **83.3%** | **87.3%** | **82.7%** | 90.5% |
|  | Regression | 83.7% | 76.7% | 78.4% | 80.8% | 80.0% | 70.5% |
| All | SVM | ^N^92.3% | ^N^92.2% | 90.1% | 89.5% | **^N^87.6%** | **96.8%** |
|  | LDA | 78.8% | 66.5% | 77.3% | 66.9% | 79.5% | 66.8% |
|  | EDT | 83.6% | 78.6% | 83.9% | 87.4% | 79.0 % | 93.2% |
|  | Regression | **^N^96.2%** | **^N^96.3%** | **93.9%** | **^N^95.5%** | ^N^87.1% | ^N^93.5% |
